# Daratumumab plus bortezomib, melphalan, and prednisone in East Asian patients with non-transplant multiple myeloma: subanalysis of the randomized phase 3 ALCYONE trial

**DOI:** 10.1007/s00277-019-03794-9

**Published:** 2019-10-16

**Authors:** Tomoaki Fujisaki, Takayuki Ishikawa, Hiroyuki Takamatsu, Kenshi Suzuki, Chang-Ki Min, Jae Hoon Lee, Jianping Wang, Robin Carson, Wendy Crist, Ming Qi, Koji Nagafuji

**Affiliations:** 1 Department of Internal Medicine, Matsuyama Red-Cross Hospital, Ehime, Japan; 2 Department of Hematology, Kobe City Medical Center General Hospital, Kobe, Japan; 3 Hematology/Respiratory Medicine, Faculty of Medicine, Institute of Medical, Pharmaceutical and Health Sciences, Kanazawa University, Kanazawa, Japan; 4 Department of Hematology, Japanese Red Cross Medical Center, Tokyo, Japan; 5 Seoul St. Mary’s Hospital, Seoul, South Korea; 6 Gachon University Gil Medical Center, Incheon, South Korea; 7 Janssen Research & Development, Raritan, NJ USA; 8 Janssen Research & Development, Spring House, PA USA; 9 Division of Hematology and Oncology, Department of Medicine, Kurume University School of Medicine, 67 Asahi-machi, Kurume, 830-0011 Japan

**Keywords:** Multiple myeloma, Daratumumab, VMP, Transplant-ineligible

## Abstract

In the ALCYONE trial, daratumumab plus bortezomib, melphalan, and prednisone (D-VMP) reduced the risk of disease progression or death by 50% versus bortezomib, melphalan, and prednisone (VMP) in patients with transplant-ineligible newly diagnosed multiple myeloma. Here, we report a subanalysis of East Asian patients from ALCYONE. After a median follow-up of 17.1 and 15.9 months for Japanese (*n* = 50) and Korean (*n* = 41) patients, respectively, median progression-free survival for D-VMP versus VMP was not reached (NR) versus 20.7 months in Japanese patients and NR versus 14.0 months in Korean patients. The overall response rate for D-VMP versus VMP was 96% versus 92% in Japanese patients and 91% versus 61% in Korean patients. Using next-generation sequencing, minimal residual disease negativity at 10^−5^ sensitivity for D-VMP versus VMP was 33% versus 8% among Japanese patients and 17% versus 0% among Korean patients. Rates of any grade and grade 3/4 pneumonia were consistent with the rates observed for the global safety population. Similar efficacy and safety findings were observed in the combined Japanese and Korean subgroup and ≥ 75 years of age subgroup. In conclusion, D-VMP was safe and efficacious in East Asian patients, consistent with the global ALCYONE population.

**Electronic supplementary material:**

The online version of this article (10.1007/s00277-019-03794-9) contains supplementary material, which is available to authorized users.

## Introduction

Daratumumab, a human IgG1 monoclonal antibody that binds CD38, exerts both on-tumor and immunomodulatory mechanisms of action against multiple myeloma cells [1–6]. Based on the results of several pivotal trials in relapsed or refractory multiple myeloma (RRMM) [7–10], daratumumab has been approved in many countries as a monotherapy and in combination with standard-of-care regimens for the treatment of RRMM [11, 12]. In Japan, daratumumab has been approved for use in combination with lenalidomide and dexamethasone or bortezomib and dexamethasone for the treatment of adults with RRMM [13].

Bortezomib, melphalan, and prednisone (VMP) was approved based on the VISTA trial in non-transplant newly diagnosed multiple myeloma (NDMM) patients and remains as one of several standard-of-care regimens for non-transplant NDMM [14]. The randomized, open-label, phase 3 ALCYONE study compared the efficacy and safety of daratumumab in combination with VMP (D-VMP) versus VMP alone in patients with NDMM who were considered ineligible for autologous stem cell transplantation (ASCT) [15]. Interim results that were reported after a median follow-up of 16.5 months showed a significant improvement in the primary endpoint of progression-free survival (PFS) with D-VMP versus VMP, along with significant improvements in response and minimal residual disease (MRD)–negativity rates at a 10^−5^ sensitivity threshold. Overall, D-VMP was associated with a toxicity profile similar to that of VMP; while grade 3 and 4 infections were more commonly seen with D-VMP relative to VMP alone, rates of discontinuations due to infection were comparable (0.9% vs 1.4% for D-VMP and VMP, respectively). Based on these results, D-VMP has been approved in the USA, European Union, and Brazil for the treatment of patients with NDMM who are ineligible for ASCT [12].

In the phase 3 POLLUX study of lenalidomide and dexamethasone (Rd) alone or with daratumumab in patients with RRMM, a subgroup analysis of Japanese, Korean, and Taiwanese participants demonstrated that daratumumab in combination with Rd conferred longer PFS and increased the rate of deeper responses versus Rd alone, consistent with the findings in the global POLLUX study population [16, 17]. To characterize the efficacy and safety profile of D-VMP versus VMP in East Asian patients with NDMM who were ineligible for ASCT, we performed a post hoc sub-analysis of East Asian (Japanese and Korean) participants enrolled into ALCYONE. Additional analyses looking at the impact of age (≥ 75 and ≥ 80 years of age) on efficacy and safety of D-VMP were also conducted.

## Methods

### Patients

A total of 91 East Asian patients from the phase 3 ALCYONE clinical trial were included in this analysis (ClinicalTrials.gov identifier: NCT02195479). Study design, patient eligibility, treatment schedule, and statistical analyses were previously published [15]; in brief, patients with newly diagnosed, documented multiple myeloma who were ≥ 65 years of age or were ineligible for high-dose chemotherapy with ASCT due to concurrent comorbidities were eligible for study inclusion. In addition, Eastern Cooperative Oncology Group (ECOG) performance status of 0 to 2 was a prerequisite for study inclusion. Hematologic, renal, and hepatic function examinations were required to be within defined limits. Patients with primary amyloidosis, monoclonal gammopathy of undetermined significance, smoldering multiple myeloma, or Waldenstrom’s macroglobulinemia were excluded. Additional exclusion criteria consisted of: prior systemic therapy or ASCT, cancer within the prior 3 years other than defined exceptions, grade ≥ 2 peripheral neuropathy, and grade ≥ 2 neuropathic pain.

### Dosing

Patients were randomized 1:1 to either D-VMP or VMP treatment groups. Up to 9 cycles of VMP (bortezomib 1.3 mg/m^2^ subcutaneously twice weekly in cycle 1, weekly in cycles 2–9; melphalan 9 mg/m^2^ orally on days 1–4 in cycles 1–9; prednisone 60 mg/m^2^ orally on days 1–4 in cycles 1–9) were administered [15]. Daratumumab was dosed at 16 mg/kg intravenously (weekly [cycle 1], every 3 weeks [cycles 2–9], and every 4 weeks thereafter until disease progression), with oral or intravenous dexamethasone administered to manage infusion-related reactions. Infusions were paused for grade ≤ 2 infusion reactions and restarted at half the original rate at the investigator’s discretion**.** Grade 4 reactions necessitated discontinuation of daratumumab, whereas infusions could be stopped for grade 3 reactions and restarted after resolution to grade 1 in severity (with discontinuation required if the event returned to grade 3 for the third time after stopping and restarting the infusion twice).

### Evaluation and statistical analyses

Analysis populations in ALCYONE included intent-to-treat (ITT) and safety populations. The ITT population was defined as all patients randomized into the study. This population was used for all primary efficacy endpoints, key secondary endpoints, and all analyses of disposition, demographic, and baseline disease characteristics. The safety population included all treated patients who had at least 1 administration of study drug and was used for all safety analyses and all analyses of treatment compliance and exposure. East Asian patients from the ITT and safety populations of ALCYONE were used for this report.

Responses were evaluated using International Myeloma Working Group criteria [18, 19]. PFS, the primary endpoint of ALCYONE**,** was evaluated using a stratified Cox regression model and stratified log-rank test. MRD, at a sensitivity threshold of 10^−5^, was assessed using bone marrow aspirate samples by next-generation sequencing using clonoSEQ^®^ version 2.0 (Adaptive Biotechnologies, Seattle, WA, USA)**.** MRD-negativity rate was defined in the study as the proportion of the ITT patients who had negative MRD assessment at any time point after randomization. If the bone marrow samples tested were MRD positive, ambiguous, or were not tested, the patient was considered MRD positive**.** High cytogenetic risk was defined as being positive for 1 or more of the following abnormalities by fluorescence in situ hybridization/karyotype: del17p, t(14;16), or t(4;14). The ITT population was used to evaluate time to best response, while responders within the ITT population were used for duration of and time to response analysis.

## Results

### Patients and treatment

The clinical cutoff date for this analysis was June 12, 2017. East Asian patients comprised 91 of 706 patients (D-VMP, *n* = 47; VMP, *n* = 44) randomized in ALCYONE, including 50 Japanese patients (D-VMP, *n* = 24; VMP, *n* = 26) and 41 Korean patients (D-VMP, *n* = 23; VMP, *n* = 18). Patient demographic and baseline disease characteristics are presented in Table 1. In East Asian patients, the median age was 71 (range, 64–93) years and 15 patients (17%) had high cytogenetic risk (9 [20%] D-VMP vs 6 [14%] of VMP patients). Three (13%) Japanese D-VMP patients and 8 (31%) Japanese VMP patients discontinued treatment, and the most common reason for treatment discontinuation was due to adverse events (AEs; 8% vs 19%, respectively). Likewise, 6 (26%) Korean patients who received D-VMP versus 12 (67%) Korean patients who received VMP discontinued treatment, and the most common reason for treatment discontinuation was due to AEs (9% vs 17%, respectively). The median (range) number of D-VMP and VMP cycles received was 14 (1–21) and 9 (1–9) for Japanese patients and 13 (1–20) and 6 (1–9) for Korean patients, respectively. The relative dose intensity of daratumumab received was 98.3% in Japanese patients and 99.6% in Korean patients. In Japanese patients, the median cumulative bortezomib dose was 50.2 mg/m^2^ in the D-VMP arm and 47.3 mg/m^2^ in the VMP arm. In Korean patients, corresponding values were 39.6 mg/m^2^ and 31.7 mg/m^2^, respectively. A detailed breakdown of cumulative exposure to bortezomib, melphalan, and prednisone by age among Japanese and Korean patients is summarized in Supplementary Table 1 in Online Resource.

**Table 1:** Demographic and baseline disease characteristics

	ALCYONE ITT population^15^		East Asian		Japanese		Korean	
	D-VMP (*n* = 350)	VMP (*n* = 356)	D-VMP (*n* = 47)	VMP (*n* = 44)	D-VMP (*n* = 24)	VMP (*n* = 26)	D-VMP (*n* = 23)	VMP (*n* = 18)
Age, *n* (%)								
< 65	36 (10.3)	24 (6.7)	1 (2.1)	0	0	0	1 (4.3)	0
65 to < 75	210 (60.0)	225 (63.2)	31 (66.0)	33 (75.0)	14 (58.3)	18 (69.2)	17 (73.9)	15 (83.3)
≥ 75	104 (29.7)	107 (30.1)	15 (31.9)	11 (25.0)	10 (41.7)	8 (30.8)	5 (21.7)	3 (16.7)
≥ 80	33 (9.4)	32 (9.0)	6 (12.8)	2 (4.5)	6 (25.0)	2 (7.7)	0	0
Median (range)	71 (40–93)	71 (50–91)	72 (64–93)	71 (66–84)	74 (65–93)	71 (66–84)	72 (64–79)	70 (66–78)
Sex, *n* (%)								
Male	160 (45.7)	167 (46.9)	25 (53.2)	18 (40.9)	13 (54.2)	12 (46.2)	12 (52.2)	6 (33.3)
Baseline ECOG score, *n* (%)								
0	78 (22.3)	99 (27.8)	16 (34.0)	16 (36.4)	15 (62.5)	11 (42.3)	1 (4.3)	5 (27.8)
1	182 (52.0)	173 (48.6)	24 (51.1)	16 (36.4)	2 (8.3)	5 (19.2)	22 (95.7)	11 (61.1)
2	90 (25.7)	84 (23.6)	7 (14.9)	12 (27.3)	7 (29.2)	10 (38.5)	0	2 (11.1)
ISS staging, *n* (%)^a^								
I	69 (19.7)	67 (18.8)	9 (19.1)	11 (25.0)	4 (16.7)	7 (26.9)	5 (21.7)	4 (22.2)
II	139 (39.7)	160 (44.9)	23 (48.9)	20 (45.5)	12 (50.0)	15 (57.7)	11 (47.8)	5 (27.8)
III	142 (40.6)	129 (36.2)	15 (31.9)	13 (29.5)	8 (33.3)	4 (15.4)	7 (30.4)	9 (50.0)
Type of myeloma, *n* (%)								
IgG	224 (64.0)	229 (64.3)	29 (61.7)	32 (72.7)	15 (62.5)	18 (69.2)	14 (60.9)	14 (77.8)
IgA	73 (20.9)	82 (23.0)	8 (17.0)	6 (13.6)	5 (20.8)	4 (15.4)	3 (13.0)	2 (11.1)
IgD	7 (2.0)	2 (0.6)	4 (8.5)	2 (4.5)	1 (4.2)	2 (7.7)	3 (13.0)	0
Light chain	36 (10.3)	33 (9.3)	6 (12.8)	4 (9.1)	3 (12.5)	2 (7.7)	3 (13.0)	2 (11.1)
Kappa	23 (6.6)	17 (4.8)	4 (8.5)	4 (9.1)	3 (12.5)	2 (7.7)	1 (4.3)	2 (11.1)
Lambda	13 (3.7)	16 (4.5)	2 (4.3)	0	0	0	2 (8.7)	0
Cytogenetics profile^b^								
*N*	314	302	45	44	24	26	21	18
Standard risk	261 (83.1)	257 (85.1)	36 (80.0)	38 (86.4)	17 (70.8)	24 (92.3)	19 (90.5)	14 (77.8)
High risk^c^	53 (16.9)	45 (14.9)	9 (20.0)	6 (13.6)	7 (29.2)	2 (7.7)	2 (9.5)	4 (22.2)
del17p	29 (9.2)	27 (8.9)	4 (8.9)	3 (6.8)	4 (16.7)	2 (7.7)	0	1 (5.6)
t(4;14)	25 (8.0)	17 (5.6)	5 (11.1)	3 (6.8)	3 (12.5)	1 (3.8)	2 (9.5)	2 (11.1)
t(14;16)	6 (1.9)	6 (2.0)	1 (2.2)	1 (2.3)	1 (4.2)	0	0	1 (5.6)
Time since initial diagnosis (months)								
Median (range)	0.76 (0.1–11.4)	0.82 (0.1–25.3)	0.59 (0.3–4.3)	0.66 (0.2–3.4)	0.82 (0.3–4.3)	0.89 (0.2–3.4)	0.49 (0.3–0.8)	0.44 (0.2–2.0)

*ITT* intent-to-treat, *D-VMP* daratumumab/bortezomib/melphalan/prednisone, *VMP* bortezomib/melphalan/prednisone, *N/A* not available, *ECOG* Eastern Cooperative Oncology Group, *ISS* International Staging System

^a^ISS staging is derived based on the combination of serum β_2_-microglobulin and albumin

^b^Cytogenetic risk is based on fluorescence in situ hybridization or karyotype testing

^c^Patient may have had ≥ 1 high-risk abnormality [del17p, t(4;14), or t(14;16)]

### Efficacy

**Fig. 1:**
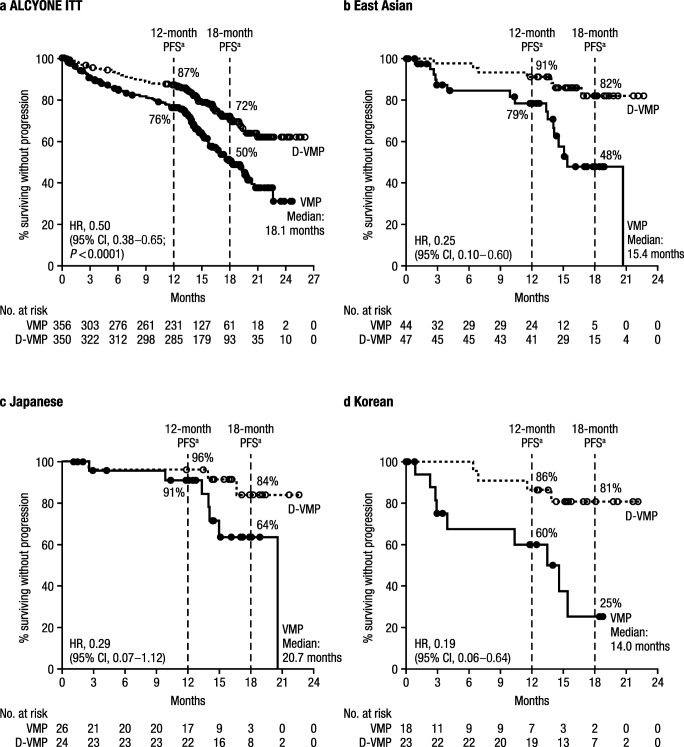
PFS of **a** ITT population, **b** East Asian patients, **c** Japanese patients, and **d** Korean patients in ALCYONE. *PFS* progression-free survival, *ITT* intent-to-treat, *D-VMP* daratumumab/bortezomib/ melphalan/prednisone, *VMP* bortezomib/melphalan/prednisone, *HR* hazard ratio, *CI* confidence interval. ^a^Kaplan-Meier estimates. Figure 1a is adapted from the *New England Journal of Medicine*. Mateos MV, et al. “Daratumumab plus Bortezomib, Melphalan, and Prednisone for Untreated Myeloma”. 378(6):518–528. Copyright © (2018) Massachusetts Medical Society. Reprinted with permission from Massachusetts Medical Society

In the combined Japanese and Korean population, the median (range) duration of follow-up was 16.9 (0.2–23.4) months. Among the individual subgroups, the median (range) duration of follow-up (based on Kaplan-Meier product-limit estimate) was 17.1 (1.1–23.4) months in Japanese patients and 15.9 (0.2–0.22) months in Korean patients. Consistent with the results in the global ITT population (Fig. 1a) [15], the median PFS in all East Asian patients was prolonged with D-VMP (not reached [NR] vs 15.4 months; hazard ratio [HR], 0.25; 95% confidence interval [CI], 0.10–0.60); the 18-month PFS rate for D-VMP versus VMP was 82% (95% CI, 65–91) versus 48% (95% CI, 27–66; Fig. 1b). Likewise, the median PFS for D-VMP versus VMP was NR versus 20.7 months in Japanese patients (HR, 0.29; 95% CI, 0.07–1.12; Fig. 1c) and NR versus 14.0 months in Korean patients (HR, 0.19; 95% CI, 0.06–0.64; Fig. 1d). The 18-month PFS rate for D-VMP versus VMP was 84% (95% CI, 55–95) versus 64% (95% CI, 35–82) in Japanese patients and 81% (95% CI, 56–92) and 25% (95% CI, 4–54) in Korean patients, respectively.

The overall response rate (ORR) in East Asian patients was higher for D-VMP (94%) versus VMP (80%), which consisted of higher rates of complete response (CR) or better (49% vs 21%) and very good partial response (VGPR) or better (81% vs 52%; Table 2). ORR, ≥ CR, and ≥ VGPR among East Asian patients treated with D-VMP were also comparable to the global ITT population receiving D-VMP (91%, 43%, and 71%, respectively) [15]. ORR for D-VMP versus VMP was 96% versus 92% in Japanese patients and 91% versus 61% in Korean patients, respectively (Table 2). In Japanese patients, the rates of CR or better and VGPR or better with D-VMP were 54% and 79%, respectively, both higher than those with VMP (23% and 62%, respectively). Korean patients also achieved both higher CR or better (44% vs 17%) and VGPR or better (83% vs 39%) rates with D-VMP compared with VMP.

**Table 2:** Overall best confirmed responses

	ALCYONE ITT population^15^		East Asian		Japanese		Korean	
	D-VMP (*n* = 350)	VMP (n = 356)	D-VMP (*n* = 47)	VMP (*n* = 44)	D-VMP (*n* = 24)	VMP (*n* = 26)	D-VMP (*n* = 23)	VMP (*n* = 18)
Response category, *n* (%)								
ORR	318 (90.9)	263 (73.9)	44 (93.6)	35 (79.5)	23 (95.8)	24 (92.3)	21 (91.3)	11 (61.1)
sCR	63 (18.0)	25 (7.0)	16 (34.0)	6 (13.6)	12 (50.0)	5 (19.2)	4 (17.4)	1 (5.6)
CR	86 (24.6)	62 (17.4)	7 (14.9)	3 (6.8)	1 (4.2)	1 (3.8)	6 (26.1)	2 (11.1)
VGPR	100 (28.6)	90 (25.3)	15 (31.9)	14 (31.8)	6 (25.0)	10 (38.5)	9 (39.1)	4 (22.2)
PR	69 (19.7)	86 (24.2)	6 (12.8)	12 (27.3)	4 (16.7)	8 (30.8)	2 (8.7)	4 (22.2)
SD	20 (5.7)	76 (21.3)	2 (4.3)	7 (15.9)	1 (4.2)	2 (7.7)	1 (4.3)	5 (27.8)
PD	0	2 (0.6)	0	0	0	0	0	0
NE	12 (3.4)	15 (4.2)	1 (2.1)	2 (4.5)	0	0	1 (4.3)	2 (11.1)
CR or better	149 (42.6)	87 (24.4)	23 (48.9)	9 (20.5)	13 (54.2)	6 (23.1)	10 (43.5)	3 (16.7)
VGPR or better	249 (71.1)	177 (49.7)	38 (80.9)	23 (52.3)	19 (79.2)	16 (61.5)	19 (82.6)	7 (38.9)

*ITT* intent-to-treat, *D-VMP* daratumumab/bortezomib/melphalan/prednisone, *VMP* bortezomib/melphalan/prednisone, *ORR* overall response rate, *sCR* stringent complete response, *CR* complete response, *VGPR* very good partial response, *PR* partial response, *SD* stable disease, *PD* progressive disease, *NE* not evaluable

In East Asian patients in the D-VMP arm versus the VMP arm, median (range) time to best response was 5.3 (0.7–15.7) versus 3.5 (0.8–14.4) months, and median duration of response was not estimable (NE) versus 19.9 (95% CI, 13.9–19.9) months. This is similar to the global ITT population that had a median time to best response of 4.9 months and 4.1 months with D-VMP and VMP, respectively, and a median duration of response that was not reached with D-VMP versus 21.3 (95% CI, 18.4—NE) months with VMP [15]. Among the subgroups, the median (range) time to best response for D-VMP versus VMP was 8.1 (0.8–15.7) months versus 3.5 (0.8–14.4) months in Japanese patients and 4.8 (0.7–14.4) months versus 3.6 (1.4–13.0) months in Korean patients. The median duration of response for D-VMP versus VMP was NE versus 19.9 (95% CI, 14.4–19.9) months in Japanese patients and NE versus 13.9 (95% CI, 9.6—NE) months in Korean patients.

Consistent with the observed response rates and with the global ITT population, the MRD-negative rate (at a 10^−5^ sensitivity threshold) was 26% for D-VMP versus 5% for VMP among all of the East Asian patients. Similar MRD-negative rates were observed among Japanese (33% vs 8%) and Korean (17% vs 0%) patients.

An exploratory subanalysis was conducted to assess the efficacy of D-VMP versus VMP using different age cutoffs in Japanese and Korean patients. In Japanese patients ≥ 75 years of age (D-VMP, *n* = 8; VMP, *n* = 10), the PFS benefit that was observed in all Japanese patients was maintained in these patients (18-month PFS: 72.0% vs 55.6% for D-VMP vs VMP, respectively; Supplementary Table 2 in Online Resource). Although fewer Korean patients ≥ 75 years of age were enrolled (D-VMP, *n* = 5; VMP, *n* = 3), a consistent PFS benefit was observed (18-month PFS: 60.0% vs 0% for D-VMP vs VMP, respectively; Supplementary Table 2 in Online Resource). Similar trends favoring D-VMP over VMP were reported for response rates and MRD-negative rates in Japanese and Korean patients ≥ 75 years of age (Supplementary Table 2 in Online Resource).

In Japanese patients ≥ 80 years of age (D-VMP, *n* = 6; VMP, *n* = 2), all patients responded to treatment, with CR or better rate being 67% versus 0%, respectively. Among these patients, the MRD-negativity rate at a 10^−5^ sensitivity threshold was also 67% versus 0%, respectively. None of the Korean patients were ≥ 80 years of age.

### Safety

The most common (> 20%) all-grade treatment-emergent adverse events (TEAEs) in East Asian patient groups are presented in Table 3**.** East Asian patients reported a higher incidence of thrombocytopenia (66% vs 57%), leukopenia (36% vs 32%), and lymphopenia (32% vs 23%) with D-VMP and VMP than the global ALCYONE safety population (thrombocytopenia, 49% vs 54%; leukopenia, 13% vs 15%; lymphopenia, 11% vs 10%) [15]. The occurrence of these cytopenias was the highest among Japanese patients treated with D-VMP (thrombocytopenia, 75%; leukopenia, 71%; and lymphopenia, 63%). In Japanese patients, the incidence of peripheral neuropathy was 29% with D-VMP versus 23% with VMP; in Korean patients, the incidence was 52% versus 67%, respectively. Rates of all-grade infection were 75% versus 69% in Japanese patients and 87% versus 50% in Korean patients; all-grade pneumonia rates were 17% versus 4% in Japanese patients and 17% versus 0% in Korean patients, consistent with rates observed in the global safety population of ALCYONE (15% vs 5%, respectively).

**Table 3:** Most common (> 20%) TEAEs

	ALCYONE safety population^15^		East Asian		Japanese		Korean	
	D-VMP (*n* = 346)	VMP (*n* = 354)	D-VMP (*n* = 47)	VMP (*n* = 44)	D-VMP (*n* = 24)	VMP (*n* = 26)	D-VMP (*n* = 23)	VMP (*n* = 18)
Hematologic, *n (%)*								
Thrombocytopenia	169 (48.8)	190 (53.7)	31 (66.0)	25 (56.8)	18 (75.0)	16 (61.5)	13 (56.5)	9 (50.0)
Neutropenia	172 (49.7)	186 (52.5)	27 (57.4)	23 (52.3)	15 (62.5)	16 (61.5)	12 (52.2)	7 (38.9)
Leukopenia	46 (13.3)	53 (15.0)	17 (36.2)	14 (31.8)	17 (70.8)	13 (50.0)	0	1 (5.6)
Lymphopenia	37 (10.7)	36 (10.2)	15 (31.9)	10 (22.7)	15 (62.5)	10 (38.5)	0	0
Anemia	97 (28.0)	133 (37.6)	11 (23.4)	14 (31.8)	4 (16.7)	7 (26.9)	7 (30.4)	7 (38.9)
Nonhematologic, *n (%)*								
Diarrhea	82 (23.7)	87 (24.6)	22 (46.8)	20 (45.5)	11 (45.8)	12 (46.2)	11 (47.8)	8 (44.4)
Pyrexia	80 (23.1)	74 (20.9)	21 (44.7)	22 (50.0)	10 (41.7)	18 (69.2)	11 (47.8)	4 (22.2)
Constipation	63 (18.2)	65 (18.4)	21 (44.7)	15 (34.1)	7 (29.2)	9 (34.6)	14 (60.9)	6 (33.3)
Decreased appetite	40 (11.6)	46 (13.0)	19 (40.4)	17 (38.6)	8 (33.3)	12 (46.2)	11 (47.8)	5 (27.8)
Peripheral sensory neuropathy	98 (28.3)	121 (34.2)	19 (40.4)	18 (40.9)	7 (29.2)	6 (23.1)	12 (52.2)	12 (66.7)
Nausea	72 (20.8)	76 (21.5)	18 (38.3)	15 (34.1)	12 (50.0)	11 (42.3)	6 (26.1)	4 (22.2)
Upper respiratory tract infection	91 (26.3)	49 (13.8)	17 (36.2)	8 (18.2)	4 (16.7)	4 (15.4)	13 (56.5)	4 (22.2)
Vomiting	59 (17.1)	55 (15.5)	12 (25.5)	6 (13.6)	9 (37.5)	4 (15.4)	3 (13.0)	2 (11.1)
Insomnia	26 (7.5)	32 (9.0)	11 (23.4)	12 (27.3)	9 (37.5)	9 (34.6)	2 (8.7)	3 (16.7)
Cough	52 (15.0)	27 (7.6)	11 (23.4)	2 (4.5)	1 (4.2)	2 (7.7)	10 (43.5)	0
Fatigue	48 (13.9)	51 (14.4)	10 (21.3)	7 (15.9)	1 (4.2)	3 (11.5)	9 (39.1)	4 (22.2)
Rash	29 (8.4)	39 (11.0)	9 (19.1)	10 (22.7)	4 (16.7)	7 (26.9)	5 (21.7)	3 (16.7)
Back pain	48 (13.9)	42 (11.9)	9 (19.1)	2 (4.5)	2 (8.3)	2 (7.7)	7 (30.4)	0
Nasopharyngitis	19 (5.5)	20 (5.6)	8 (17.0)	7 (15.9)	6 (25.0)	6 (23.1)	2 (8.7)	1 (5.6)
Dyspepsia	18 (5.2)	12 (3.4)	8 (17.0)	4 (9.1)	1 (4.2)	2 (7.7)	7 (30.4)	2 (11.1)
Injection-site erythema	12 (3.5)	28 (7.9)	7 (14.9)	14 (31.8)	7 (29.2)	13 (50.0)	0	1 (5.6)
Viral upper respiratory tract infection	14 (4.0)	3 (0.8)	7 (14.9)	1 (2.3)	5 (20.8)	1 (3.8)	2 (8.7)	0
Chills	26 (7.5)	6 (1.7)	7 (14.9)	2 (4.5)	2 (8.3)	0	5 (21.7)	2 (11.1)
Peripheral edema	62 (17.9)	39 (11.0)	6 (12.8)	4 (9.1)	6 (25.0)	3 (11.5)	0	1 (5.6)
Increased ALT	15 (4.3)	18 (5.1)	6 (12.8)	6 (13.6)	1 (4.2)	4 (15.4)	5 (21.7)	2 (11.1)
Increased AST	13 (3.8)	15 (4.2)	5 (10.6)	6 (13.6)	0	4 (15.4)	5 (21.7)	2 (11.1)
Malaise	7 (2.0)	10 (2.8)	3 (6.4)	6 (13.6)	3 (12.5)	6 (23.1)	0	0

*TEAE* treatment-emergent adverse event, *D-VMP* daratumumab/bortezomib/melphalan/prednisone, *VMP* bortezomib/melphalan/prednisone, *ALT* alanine aminotransferase, *AST* aspartate aminotransferase

The most common (> 10%) grade 3 or 4 TEAEs for East Asian patient groups are summarized in Table 4. Similar to all-grade TEAEs, the incidence of grade 3/4 cytopenias was higher among East Asian patients with D-VMP and VMP (neutropenia, 55% vs 52%; thrombocytopenia, 55% vs 46%; leukopenia, 32% vs 27%; lymphopenia, 32% vs 18%; anemia, 17% vs 25%) as compared to the global ALCYONE safety population (neutropenia, 40% vs 39%; thrombocytopenia, 34% vs 38%; leukopenia, 8% vs 9%; lymphopenia, 8% vs 6%; anemia, 16% vs 20%); however, no East Asian patients discontinued treatment due to any type of cytopenia [15]. Japanese patients had the highest occurrence of grade 3/4 cytopenias with D-VMP and VMP including neutropenia (58% vs 62%), thrombocytopenia (58% vs 46%), leukopenia (63% vs 42%), and lymphopenia (63% vs 31%).

**Table 4:** Most common (> 10%) grade 3 and 4 TEAEs

	ALCYONE safety population^15^		East Asian		Japanese		Korean	
	D-VMP (*n* = 346)	VMP (*n* = 354)	D-VMP (*n* = 47)	VMP (*n* = 44)	D-VMP (*n* = 24)	VMP (*n* = 26)	D-VMP (*n* = 23)	VMP (*n* = 18)
Hematologic, *n* (%)								
Neutropenia	138 (39.9)	137 (38.7)	26 (55.3)	23 (52.3)	14 (58.3)	16 (61.5)	12 (52.2)	7 (38.9)
Thrombocytopenia	119 (34.4)	133 (37.6)	26 (55.3)	20 (45.5)	14 (58.3)	12 (46.2)	12 (52.2)	8 (44.4)
Leukopenia	28 (8.1)	30 (8.5)	15 (31.9)	12 (27.3)	15 (62.5)	11 (42.3)	0	1 (5.6)
Lymphopenia	26 (7.5)	22 (6.2)	15 (31.9)	8 (18.2)	15 (62.5)	8 (30.8)	0	0
Anemia	55 (15.9)	70 (19.8)	8 (17.0)	11 (25.0)	2 (8.3)	6 (23.1)	6 (26.1)	5 (27.8)
Nonhematologic, *n* (%)								
Pneumonia	39 (11.3)	14 (4.0)	6 (12.8)	0	2 (8.3)	0	4 (17.4)	0
Increased ALT	6 (1.7)	5 (1.4)	4 (8.5)	2 (4.5)	1 (4.2)	0	3 (13.0)	2 (11.1)
Fatigue	11 (3.2)	9 (2.5)	4 (8.5)	3 (6.8)	0	1 (3.8)	4 (17.4)	2 (11.1)
Hyponatremia	8 (2.3)	9 (2.5)	3 (6.4)	3 (6.8)	3 (12.5)	1 (3.8)	0	2 (11.1)
Diarrhea	9 (2.6)	11 (3.1)	3 (6.4)	3 (6.8)	2 (8.3)	1 (3.8)	1 (4.3)	2 (11.1)
Increased AST	5 (1.4)	4 (1.1)	2 (4.3)	2 (4.5)	0	0	2 (8.7)	2 (11.1)
Hypertension	14 (4.0)	6 (1.7)	2 (4.3)	2 (4.5)	0	0	2 (8.7)	2 (11.1)
Asthenia	4 (1.2)	7 (2.0)	1 (2.1)	2 (4.5)	0	0	1 (4.3)	2 (11.1)
Increased C-reactive protein	2 (0.6)	2 (0.6)	0	2 (4.5)	0	0	0	2 (11.1)
Septic shock	1 (0.3)	3 (0.8)	0	2 (4.5)	0	0	0	2 (11.1)

*TEAE* treatment-emergent adverse event, *D-VMP* daratumumab/bortezomib/melphalan/prednisone, *VMP* bortezomib/melphalan/prednisone, *ALT* alanine aminotransferase, *AST* aspartate aminotransferase

The incidence of grade 3 or 4 infections among East Asian patients was 19% with D-VMP (no grade 4 events) versus 16% with VMP (6 grade 3 events; 1 grade 4 event). In Japanese patients, the incidence of grade 3 infections was 21% with D-VMP versus 12% with VMP (no grade 4 events observed in either arm); in Korean patients, grade 3 or 4 infections were reported in 19% of D-VMP patients (all grade 3) versus 16% of VMP patients (all grade 3 except for 1 patient with grade 4 septic shock). Regarding discontinuations due to TEAEs, these occurred in 9% and 18% of all East Asian patients, and 8% and 19% of Japanese patients receiving D-VMP and VMP, respectively; in Korean patients, they occurred in 9% and 17% of patients receiving D-VMP and VMP, respectively. None of the Japanese patients receiving D-VMP discontinued treatment due to pneumonia or any other infections, whereas 2 Japanese patients receiving VMP discontinued treatment due to infections (pneumonia and tuberculosis pleurisy). Among Korean patients, 1 patient discontinued due to infection each in the D-VMP (pneumonia) and VMP (pelvic infection) treatment groups.

Daratumumab-associated infusion reactions were reported by 40% of all East Asian patients, which was higher than that reported in the global safety population (28%). Daratumumab-associated infusion reactions were reported by 42% of Japanese patients and 39% of Korean patients. Infusion reactions occurred during the first infusions in all but 1 patient in each cohort. No patients discontinued treatment due to infusion reactions.

A total of 2 East Asian patients reported second primary malignancies (SPMs). Among Japanese patients, no patients receiving D-VMP reported SPMs; 1 patient receiving VMP developed gastric adenocarcinoma. Among Korean patients, 1 patient receiving D-VMP reported renal cell carcinoma; no patients in the VMP arm reported SPMs.

Similar safety findings were observed among Japanese and Korean patients ≥ 75 years of age (Supplementary Table 3 in Online Resource). For D-VMP versus VMP, grade 3 infections occurred in 4 (40%) versus 1 (13%) Japanese patients and in 1 (20%) versus 1 (33%) Korean patient, respectively; no grade 4 infection events were noted in Japanese or Korean patients. Discontinuations due to TEAEs were observed in 2 (20%) versus 3 (38%) Japanese patients and 1 (20%) versus 1 (33%) Korean patient, respectively; none of the discontinuations due to TEAEs in the D-VMP arm for Japanese and Korean patients were due to infections. Five (50%) Japanese patients and 1 (20%) Korean patient ≥ 75 years of age reported infusion reactions, with only one grade 3 event observed in 1 Japanese patient (dyspnea).

Among Japanese patients ≥ 80 years of age, TEAEs that occurred in > 1 patient are summarized in Supplementary Table 4 in Online Resource**.** Grade 3 infections were observed in 2 (33%) Japanese patients ≥ 80 years of age receiving D-VMP (cytomegalovirus infection, disseminated herpes zoster, influenza, and pneumonia [*n* = 1 each]) and 1 receiving VMP (50%; tuberculosis pleurisy). Two (33%) patients had infusion reactions, both of which were grades 1 or 2 in severity (nausea and decreased oxygen saturation).

## Discussion

Bortezomib-containing regimens are usually recommended for NDMM patients, with VMP being a recommended regimen for transplant-ineligible patients in Asian countries [20]. Within the ITT population of ALCYONE, a phase 3 study that evaluated D-VMP versus VMP in NDMM patients ineligible for ASCT, a significant PFS benefit was observed with the addition of daratumumab to VMP versus VMP alone (HR, 0.50; 95% CI, 0.38–0.65; *P* < 0.001), with 18-month PFS rates of 72% and 50% observed for D-VMP and VMP, respectively [15]. In addition, ORR (91% vs 74%), VGPR or better (71% vs 50%), CR or better (43% vs 24%), and MRD-negativity rates at a 10^−5^ sensitivity threshold (22% vs 6%) were all significantly improved (all *P* < 0.001) with D-VMP versus VMP alone [15]. The impact of adding daratumumab to VMP on overall survival has yet to be determined and will depend on a longer follow-up. The current efficacy findings reported here for East Asian patients are consistent with those from the global population of the ALCYONE study [15]. Comparable HRs and 18-month PFS rates were observed with D-VMP versus VMP in respective and combined Japanese and Korean subgroups, along with consistent benefit demonstrating improvements in ORR and MRD-negativity rates at a 10^−5^ sensitivity threshold for D-VMP versus VMP.

The incidences of several TEAEs were generally higher in East Asian patients for both D-VMP and VMP treatment arms compared with the global safety population, but no new safety signals were observed. A higher rate of cytopenias was reported in Japanese patients relative to the global ALCYONE ITT study population. It is unclear as to why Japanese patients experienced a higher incidence of cytopenias, but a high occurrence of thrombocytopenia and lymphopenia has been reported previously in Japanese patients with RRMM treated with daratumumab and bortezomib combination therapy [21]. Nevertheless, the number of Japanese patients in this study was small, and the rates of cytopenia were consistently elevated in both D-VMP and VMP treatment groups. In addition, the high rates of cytopenia did not result in higher rates of infection relative to the global ALCYONE study population, and no patients discontinued treatment due to cytopenias, suggesting that it was well managed.

Japanese D-VMP recipients had similar rates of infections compared to the global population, with a grade 3 incidence of 21% (compared with 23% in the global D-VMP population) [15], suggesting that infections were manageable in Japanese patients. Discontinuation rates for Japanese patients were low, with no Japanese patients who received D-VMP discontinuing due to infections. Among Korean patients, lower rates of hematologic TEAEs were observed, with the exception of anemia. In all East Asian patients, the majority of infections were mild and did not require hospitalization, and most resolved over time without resulting in treatment discontinuations. Although rates of infusion-related reactions were numerically higher in Japanese (42%) and Korean (39%) patients compared with the global population (28%) [15], these rates are consistent with rates observed across all other daratumumab studies [12]. Addition of daratumumab to VMP did not increase rates of SPMs in Japanese and Korean patients.

Safety and treatment-related toxicities are a primary concern in the management of elderly NDMM patients [22, 23]. Despite the small number of patients ≥ 75 years of age, incidences of the most common TEAEs in patients ≥ 75 years of age were generally aligned with that of the global safety population in ALCYONE. These results, together with those from phase 1 trials of daratumumab in Japanese patients and similar post hoc analyses of East Asian participants from a global phase 3 study [16, 21, 24], confirm that East Asian patients demonstrate similar tolerability to daratumumab-based regimens as compared with the global trial population.

East Asian patients demonstrated a higher stringent complete response (sCR) rate (34%) which was predominately driven by Japanese D-VMP patients achieving a 50% sCR rate. The higher sCR among East Asian patients in ALCYONE may be due in part to the relatively high cumulative exposure to bortezomib; however, these observations should be interpreted with caution due to the analyses not being statistically powered and are based on limited patient numbers in the ALCYONE subgroup analyses. The post hoc analysis performed in the current study, while lacking the statistical rigor of prespecified subgroup analyses, provides further support that East Asian patients derive a comparable efficacy benefit from daratumumab containing regimens relative to international trial populations [16]. Future studies are planned to extend these findings to survival outcomes in East Asian patients.

The contribution of ethnic background and genetic variation to the efficacy of daratumumab remains unexplored. A previous study of elotuzumab, lenalidomide, and dexamethasone (Elo-Rd) in RRMM found that patients homozygous for the high-affinity FcγRIIIa V allele had dramatically improved PFS of 22.3 months compared with 9.8 months in Elo-Rd-treated patients homozygous for the low-affinity FcγRIIIa F allele [25]. Ethnic differences in FcγRIIIa variant expression were not reported. Future studies examining receptor polymorphisms across patient cohorts will help to elucidate potential ethnic differences in patient response to daratumumab and other biologics in myeloma.

In conclusion, the addition of daratumumab to VMP was efficacious with no new safety signals in both Japanese and Korean patients. Although patient sample sizes were small, the benefit of D-VMP in East Asian patients was consistent with that of the global population of ALCYONE.

## Electronic supplementary material

ESM 1 (DOCX 21 kb)

## Data Availability

The data sharing policy of Janssen Pharmaceutical Companies of Johnson & Johnson is available at https://www.janssen.com/clinical-trials/transparency. As noted on this site, requests for access to the study data can be submitted through Yale Open Data Access (YODA) Project site at http://yoda.yale.edu.

## References

[1] de Weers M, Tai YT, van der Veer MS, Bakker JM, Vink T, Jacobs DC, Oomen LA, Peipp M, Valerius T, Slootstra JW, Mutis T, Bleeker WK, Anderson KC, Lokhorst HM, van de Winkel JG, Parren PW (2011). Daratumumab, a novel therapeutic human CD38 monoclonal antibody, induces killing of multiple myeloma and other hematological tumors. J Immunol.

[2] Lammerts van Bueren J, Jakobs D, Kaldenhoven N, Roza M, Hiddingh S, Meesters J, Voorhorst M, Gresnigt E, Wiegman L, Buijsse O, Andringa G, Overdijk MB, Doshi P, Sasser K, de Weers M, Parren PWHI (2014). Direct in vitro comparison of daratumumab with surrogate analogs of CD38 antibodies MOR03087, SAR650984 and Ab79. Blood.

[3] Overdijk Marije B, Verploegen Sandra, Bögels Marijn, van Egmond Marjolein, van Bueren Jeroen J Lammerts, Mutis Tuna, Groen Richard WJ, Breij Esther, Martens Anton CM, Bleeker Wim K, Parren Paul WHI (2015). Antibody-mediated phagocytosis contributes to the anti-tumor activity of the therapeutic antibody daratumumab in lymphoma and multiple myeloma. mAbs.

[4] van de Donk NWCJ, Janmaat ML, Mutis T, Lammerts van Bueren JJ, Ahmadi T, Sasser AK, Lokhorst HM, Parren PWHI (2016). Monoclonal antibodies targeting CD38 in hematological malignancies and beyond. Immunol Rev.

[5] Krejcik J, Casneuf T, Nijhof IS, Verbist B, Bald J, Plesner T, Syed K, Liu K, van de Donk NWCJ, Weiss BM, Ahmadi T, Lokhorst HM, Mutis T, Sasser AK (2016). Daratumumab depletes CD38^+^ immune-regulatory cells, promotes T-cell expansion, and skews T-cell repertoire in multiple myeloma. Blood.

[6] Overdijk MB, Jansen JH, Nederend M, Lammerts van Bueren JJ, Groen RW, Parren PW, Leusen JH, Boross P (2016). The therapeutic CD38 monoclonal antibody daratumumab induces programmed cell death via Fcgamma receptor-mediated cross-linking. J Immunol.

[7] Lokhorst HM, Plesner T, Laubach JP, Nahi H, Gimsing P, Hansson M, Minnema MC, Lassen U, Krejcik J, Palumbo A, van de Donk NWCJ, Ahmadi T, Khan I, Uhlar CM, Wang J, Sasser AK, Losic N, Lisby S, Basse L, Brun N, Richardson PG (2015). Targeting CD38 with daratumumab monotherapy in multiple myeloma. N Engl J Med.

[8] Lonial S, Weiss BM, Usmani SZ, Singhal S, Chari A, Bahlis NJ, Belch A, Krishnan A, Vescio RA, Mateos MV, Mazumder A, Orlowski RZ, Sutherland HJ, Blade J, Scott EC, Oriol A, Berdeja J, Gharibo M, Stevens DA, LeBlanc R, Sebag M, Callander N, Jakubowiak A, White D, de la Rubia J, Richardson PG, Lisby S, Feng H, Uhlar CM, Khan I, Ahmadi T, Voorhees PM (2016). Daratumumab monotherapy in patients with treatment-refractory multiple myeloma (SIRIUS): an open-label, randomised, phase 2 trial. Lancet.

[9] Palumbo A, Chanan-Khan A, Weisel K, Nooka AK, Masszi T, Beksac M, Spicka I, Hungria V, Munder M, Mateos MV, Mark TM, Qi M, Schecter J, Amin H, Qin X, Deraedt W, Ahmadi T, Spencer A, Sonneveld P (2016). Daratumumab, bortezomib, and dexamethasone for multiple myeloma. N Engl J Med.

[10] Dimopoulos MA, Oriol A, Nahi H, San-Miguel J, Bahlis NJ, Usmani SZ, Rabin N, Orlowski RZ, Komarnicki M, Suzuki K, Plesner T, Yoon SS, Yehuda DB, Richardson PG, Goldschmidt H, Reece D, Lisby S, Khokhar NZ, O'Rourke DM, Chiu C, Qin X, Guckert M, Ahmadi T, Moreau P (2016). Daratumumab, lenalidomide, and dexamethasone for multiple myeloma. N Engl J Med.

[11] European Medicines Agency (2018) Committee for Medicinal Products for Human Use post-authorization summary of positive opinion for Darzalex (II-11). https://www.ema.europa.eu/en/medicines/human/EPAR/darzalex. Accessed 29 March 2019

[12] DARZALEX^®^ (daratumumab) injection, for intravenous use [package insert]. Horsham, PA: Janssen Biotech, Inc; 2019

[13] Genmab (2017) Genmab announces approval of DARZALEX^®^ (daratumumab) for relapsed or refractory multiple myeloma in Japan. https://ir.genmab.com/news-releases/news-release-details/genmab-announces-approval-darzalexr-daratumumab-relapsed-or. Accessed 29 March 2019

[14] San Miguel JF, Schlag R, Khuageva NK, Dimopoulos MA, Shpilberg O, Kropff M, Spicka I, Petrucci MT, Palumbo A, Samoilova OS, Dmoszynska A, Abdulkadyrov KM, Schots R, Jiang B, Mateos MV, Anderson KC, Esseltine DL, Liu K, Cakana A, van de Velde H, Richardson PG (2008). Bortezomib plus melphalan and prednisone for initial treatment of multiple myeloma. N Engl J Med.

[15] Mateos MV, Dimopoulos MA, Cavo M, Suzuki K, Jakubowiak A, Knop S, Doyen C, Lucio P, Nagy Z, Kaplan P, Pour L, Cook M, Grosicki S, Crepaldi A, Liberati AM, Campbell P, Shelekhova T, Yoon SS, Iosava G, Fujisaki T, Garg M, Chiu C, Wang J, Carson R, Crist W, Deraedt W, Nguyen H, Qi M, San-Miguel J (2018). Daratumumab plus bortezomib, melphalan, and prednisone for untreated myeloma. N Engl J Med.

[16] Suzuki K, Dimopoulos MA, Takezako N, Okamoto S, Shinagawa A, Matsumoto M, Kosugi H, Yoon SS, Huang SY, Qin X, Qi M, Iida S (2018). Daratumumab, lenalidomide, and dexamethasone in East Asian patients with relapsed or refractory multiple myeloma: subgroup analyses of the phase 3 POLLUX study. Blood Cancer J.

[17] Dimopoulos MA, San-Miguel J, Belch A, White D, Benboubker L, Cook G, Leiba M, Morton J, Ho PJ, Kim K, Takezako N, Moreau P, Kaufman JL, Sutherland HJ, Lalancette M, Magen H, Iida S, Kim JS, Prince HM, Cochrane T, Oriol A, Bahlis NJ, Chari A, O'Rourke L, Wu K, Schecter JM, Casneuf T, Chiu C, Soong D, Sasser AK, Khokhar NZ, Avet-Loiseau H, Usmani SZ (2018). Daratumumab plus lenalidomide and dexamethasone versus lenalidomide and dexamethasone in relapsed or refractory multiple myeloma: updated analysis of POLLUX. Haematologica.

[18] Rajkumar SV, Harousseau JL, Durie B, Anderson KC, Dimopoulos M, Kyle R, Blade J, Richardson P, Orlowski R, Siegel D, Jagannath S, Facon T, Avet-Loiseau H, Lonial S, Palumbo A, Zonder J, Ludwig H, Vesole D, Sezer O, Munshi NC, San Miguel J (2011). Consensus recommendations for the uniform reporting of clinical trials: report of the International Myeloma Workshop Consensus Panel 1. Blood.

[19] Durie BGM, Harousseau JL, Miguel JS, Blade J, Barlogie B, Anderson K, Gertz M, Dimopoulos M, Westin J, Sonneveld P, Ludwig H, Gahrton G, Beksac M, Crowley J, Belch A, Boccadaro M, Cavo M, Turesson I, Joshua D, Vesole D, Kyle R, Alexanian R, Tricot G, Attal M, Merlini G, Powles R, Richardson P, Shimizu K, Tosi P, Morgan G, Rajkumar SV (2006). International uniform response criteria for multiple myeloma. Leukemia.

[20] Tan D, Lee JH, Chen W, Shimizu K, Hou J, Suzuki K, Nawarawong W, Huang SY, Sang Chim C, Kim K, Kumar L, Malhotra P, Chng WJ, Durie B; Asian Myeloma Network (2018) Recent advances in the management of multiple myeloma: clinical impact based on resource-stratification. Consensus statement of the Asian Myeloma Network at the 16th international myeloma workshop. Leuk Lymphoma 59:2305–2317 10.1080/10428194.2018.1427858 29390932

[21] Iida S, Ichinohe T, Shinagawa A, Suzuki K, Takezako N, Aoki M (2018). Safety and efficacy of daratumumab in combination with bortezomib and dexamethasone in Japanese patients with relapsed or refractory multiple myeloma. Int J Hematol.

[22] Palumbo A, Bringhen S, Mateos MV, Larocca A, Facon T, Kumar SK, Offidani M, McCarthy P, Evangelista A, Lonial S, Zweegman S, Musto P, Terpos E, Belch A, Hajek R, Ludwig H, Stewart AK, Moreau P, Anderson K, Einsele H, Durie BG, Dimopoulos MA, Landgren O, San Miguel JF, Richardson P, Sonneveld P, Rajkumar SV (2015). Geriatric assessment predicts survival and toxicities in elderly myeloma patients: an International Myeloma Working Group report. Blood.

[23] Willan J, Eyre TA, Sharpley F, Watson C, King AJ, Ramasamy K (2016). Multiple myeloma in the very elderly patient: challenges and solutions. Clin Interv Aging.

[24] Iida S, Suzuki K, Kusumoto S, Ri M, Tsukada N, Abe Y, Aoki M, Inagaki M (2017). Safety and efficacy of daratumumab in Japanese patients with relapsed or refractory multiple myeloma: a multicenter, phase 1, dose-escalation study. Int J Hematol.

[25] Jakubowiak Andrzej, Offidani Massimo, Pégourie Brigitte, De La Rubia Javier, Garderet Laurent, Laribi Kamel, Bosi Alberto, Marasca Roberto, Laubach Jacob, Mohrbacher Ann, Carella Angelo Michele, Singhal Anil K., Tsao L. Claire, Lynch Mark, Bleickardt Eric, Jou Ying-Ming, Robbins Michael, Palumbo Antonio (2016). Randomized phase 2 study: elotuzumab plus bortezomib/dexamethasone vs bortezomib/dexamethasone for relapsed/refractory MM. Blood.

