# Supplementary material for: Daratumumab plus bortezomib, melphalan, and prednisone in East Asian patients with non-transplant multiple myeloma: subanalysis of the randomized phase 3 ALCYONE trial
Source: Ann Hematol. 2019 Oct 16;98(12):2805–14. doi: 10.1007/s00277-019-03794-9 (PMC6900260; doi:10.1007/s00277-019-03794-9)
Supplement: Supplementary file 1 — (DOCX 21 kb) [file 277_2019_3794_MOESM1_ESM.docx]

**ELECTRONIC SUPPLEMENTARY MATERIAL**

**Daratumumab plus bortezomib, melphalan, and prednisone in East Asian patients with non-transplant multiple myeloma: subanalysis of the randomized phase 3 ALCYONE trial**

Annals of Hematology

Tomoaki Fujisaki, Takayuki Ishikawa, Hiroyuki Takamatsu, Kenshi Suzuki, Chang-Ki Min, Jae Hoon Lee, Jianping Wang, Robin Carson, Wendy Crist, Ming Qi, Koji Nagafuji

**Corresponding author**:

Prof. Koji Nagafuji

Division of Hematology and Oncology, Department of Medicine

Kurume University School of Medicine

67 Asahi-machi, Kurume 830-0011, Japan

Email: [knagafuji@med.kurume-u.ac.jp](mailto:knagafuji@med.kurume-u.ac.jp)

**SUPPLEMENTARY TABLES**

**Supplementary Table 1 Median Cumulative Exposure to Bortezomib, Melphalan, and Prednisone by Age**

|  | **≥75 years of age** | | | | **<75 years of age** | | | |
| --- | --- | --- | --- | --- | --- | --- | --- | --- |
|  | **Japanese** | | **Korean** | | **Japanese** | | **Korean** | |
|  | **D-VMP**  **(n = 10)** | **VMP**  **(n = 8)** | **D-VMP**  **(n = 5)** | **VMP**  **(n = 3)** | **D-VMP**  **(n = 14)** | **VMP**  **(n = 18)** | **D-VMP**  **(n = 18)** | **VMP**  **(n = 15)** |
| **Bortezomib, mg/m^2^**  Range | 39.0  1.3-51.8 | 28.3  3.9-51.1 | 24.5  16.8-51.1 | 18.5  5.1-28.9 | 51.1  44.7-53.0 | 49.0  7.2-52.4 | 39.7  2.7-53.9 | 39.5  2.6-55.0 |
| **Melphalan, mg/m^2^**  Range | 228.5  25.1-299.4 | 207.0  34.1-310.7 | 181.4  137.7-346.0 | 182.1  35.4-185.2 | 291.6  243.4-326.5 | 283.8  31.6-345.3 | 297.0  37.1-347.0 | 254.1  34.3-346.5 |
| **Prednisone, mg/m^2^**  Range | 2287.0  204.0-2440.0 | 1448.0  241.8-2219.0 | 1313.3  1119.3-2492.6 | 1164.2  240.1-1214.1 | 2331.9  2123.3-2584.6 | 2141.0  230.3-2215.3 | 2354.3  247.1-2579.8 | 1644.2  236.9-2227.3 |

D-VMP, daratumumab/bortezomib/melphalan/prednisone; VMP, bortezomib/melphalan/prednisone

**Supplementary Table 2 Summary of Efficacy in Patients ≥75 Years of Age**

|  | **Japanese** | | **Korean** | | |
| --- | --- | --- | --- | --- | --- |
|  | **D-VMP**  **(n = 10)** | **VMP**  **(n = 8)** | | **D-VMP**  **(n = 5)** | **VMP**  **(n = 3)** |
| **PFS** |  |  | |  |  |
| Median, months | NE | 20.7 | | NE | 8.7 |
| 18-month PFS, % | 72.0 | 55.6 | | 60.0 | 0 |
| **Response rate, n (%)** |  |  | |  |  |
| ORR | 9 (90.0) | 7 (87.5) | | 5 (100.0) | 1 (33.3) |
| sCR | 5 (50.0) | 1 (12.5) | | 0 | 0 |
| CR | 0 | 0 | | 0 | 0 |
| VGPR | 3 (30.0) | 2 (25.0) | | 3 (60.0) | 1 (33.3) |
| PR | 1 (10.0) | 4 (50.0) | | 2 (40.0) | 0 |
| **MRD-negativity rate at 10^–5^, n (%)** | 5 (50.0) | 1 (12.5) | | 0 | 0 |

D-VMP, daratumumab/bortezomib/melphalan/prednisone; VMP, bortezomib/melphalan/prednisone; PFS, progression-free survival; NE, not evaluable;

ORR, overall response rate; sCR, stringent complete response; CR, complete response; VGPR, very good partial response; PR, partial response;

MRD, minimal residual disease

**Supplementary Table 3 Most Common (>1 Patient) TEAEs in Patients ≥75 Years of Age**

|  | **Japanese** | | **Korean** | | |
| --- | --- | --- | --- | --- | --- |
|  | **D-VMP**  **(n = 10)** | **VMP**  **(n = 8)** | | **D-VMP**  **(n = 5)** | **VMP**  **(n = 3)** |
| **Hematologic, n (%)** |  |  | |  |  |
| Leukopenia | 9 (90.0) | 5 (62.5) | | 0 | 0 |
| Thrombocytopenia | 9 (90.0) | 4 (50.0) | | 3 (60.0) | 2 (66.7) |
| Neutropenia | 8 (80.0) | 4 (50.0) | | 2 (40.0) | 1 (33.3) |
| Lymphopenia | 7 (70.0) | 5 (62.5) | | 0 | 0 |
| Anemia | 3 (30.0) | 3 (37.5) | | 1 (20.0) | 0 |
| **Nonhematologic, n (%)** |  |  | |  |  |
| Nausea | 7 (70.0) | 4 (50.0) | | 2 (40.0) | 0 |
| Diarrhea | 6 (60.0) | 4 (50.0) | | 2 (40.0) | 2 (66.7) |
| Decreased appetite | 5 (50.0) | 7 (87.5) | | 2 (40.0) | 1 (33.3) |
| Insomnia | 5 (50.0) | 5 (62.5) | | 1 (20.0) | 0 |
| Injection-site erythema | 4 (40.0) | 5 (62.5) | | 0 | 0 |
| Pyrexia | 4 (40.0) | 5 (62.5) | | 4 (80.0) | 1 (33.3) |
| Vomiting | 4 (40.0) | 1 (12.5) | | 2 (40.0) | 0 |
| Pneumonia | 4 (40.0) | 0 | | 1 (20.0) | 0 |
| Influenza | 3 (30.0) | 1 (12.5) | | 0 | 0 |
| Malaise | 3 (30.0) | 1 (12.5) | | 0 | 0 |
| Hyponatremia | 3 (30.0) | 1 (12.5) | | 1 (20.0) | 0 |
| Nasopharyngitis | 3 (30.0) | 0 | | 1 (20.0) | 0 |
| Headache | 3 (30.0) | 0 | | 0 | 0 |
| Delirium | 3 (30.0) | 0 | | 1 (20.0) | 0 |
| Constipation | 2 (20.0) | 4 (50.0) | | 4 (80.0) | 1 (33.3) |
| Peripheral sensory neuropathy | 2 (20.0) | 2 (25.0) | | 2 (40.0) | 2 (66.7) |
| Oral candidiasis | 2 (20.0) | 2 (25.0) | | 0 | 0 |
| Peripheral edema | 2 (20.0) | 1 (12.5) | | 0 | 1 (33.3) |
| Rib fracture | 2 (20.0) | 1 (12.5) | | 0 | 0 |
| Bronchitis | 2 (20.0) | 0 | | 1 (20.0) | 1 (33.3) |
| Cytomegalovirus infection | 2 (20.0) | 0 | | 0 | 0 |
| Chills | 2 (20.0) | 0 | | 2 (40.0) | 0 |
| Urinary tract infection | 2 (20.0) | 0 | | 0 | 0 |
| Viral upper respiratory tract infection | 2 (20.0) | 0 | | 0 | 0 |
| Hypokalemia | 2 (20.0) | 0 | | 0 | 0 |
| Contusion | 2 (20.0) | 0 | | 0 | 0 |
| Spinal compression fracture | 2 (20.0) | 0 | | 0 | 0 |
| Drug eruption | 2 (20.0) | 0 | | 0 | 0 |
| Stomatitis | 1 (10.0) | 2 (25.0) | | 0 | 1 (33.3) |
| Dysgeusia | 1 (10.0) | 2 (25.0) | | 0 | 0 |
| Upper respiratory tract infection | 1 (10.0) | 1 (12.5) | | 0 | 2 (66.7) |
| Cough | 1 (10.0) | 1 (12.5) | | 3 (60.0) | 0 |
| Rash | 1 (10.0) | 1 (12.5) | | 2 (40.0) | 0 |
| Erythema multiforme | 0 | 2 (25.0) | | 0 | 0 |
| Fatigue | 0 | 2 (25.0) | | 1 (20.0) | 0 |
| Pain in extremity | 0 | 2 (25.0) | | 1 (20.0) | 1 (33.3) |
| Dyspepsia | 0 | 0 | | 2 (40.0) | 0 |
| Asthenia | 0 | 0 | | 2 (40.0) | 0 |
| Fluid retention | 0 | 0 | | 2 (40.0) | 0 |

TEAE, treatment-emergent adverse event; D-VMP, daratumumab/bortezomib/melphalan/prednisone; VMP, bortezomib/melphalan/prednisone

**Supplementary Table 4 Most Common (>1 Patient) TEAEs in Patients ≥80 Years of Age**

|  | **Japanese** | |
| --- | --- | --- |
|  | **D-VMP**  **(n = 6)** | **VMP**  **(n = 2)** |
| **Hematologic, n (%)** |  |  |
| Leukopenia | 6 (100.0) | 1 (50.0) |
| Lymphopenia | 5 (83.3) | 1 (50.0) |
| Neutropenia | 5 (83.3) | 1 (50.0) |
| Thrombocytopenia | 5 (83.3) | 0 |
| Anemia | 3 (50.0) | 0 |
| **Nonhematologic, n (%)** |  |  |
| Insomnia | 3 (50.0) | 2 (100.0) |
| Diarrhea | 3 (50.0) | 1 (50.0) |
| Nausea | 3 (50.0) | 1 (50.0) |
| Decreased appetite | 3 (50.0) | 1 (50.0) |
| Hyponatremia | 3 (50.0) | 1 (50.0) |
| Vomiting | 3 (50.0) | 0 |
| Pneumonia | 3 (50.0) | 0 |
| Injection-site erythema | 3 (50.0) | 0 |
| Pyrexia | 2 (33.3) | 1 (50.0) |
| Influenza | 2 (33.3) | 0 |
| Nasopharyngitis | 2 (33.3) | 0 |
| Viral upper respiratory tract infection | 2 (33.3) | 0 |
| Malaise | 2 (33.3) | 0 |
| Contusion | 2 (33.3) | 0 |
| Rib fracture | 2 (33.3) | 0 |
| Spinal compression fracture | 2 (33.3) | 0 |
| Delirium | 2 (33.3) | 0 |

TEAE, treatment-emergent adverse event; D-VMP, daratumumab/bortezomib/melphalan/prednisone;

VMP, bortezomib/melphalan/prednisone
